# Effect of internal contamination with tritiated water on the neoplastic colonies in the lungs, innate anti-tumour reactions, cytokine profile, and haematopoietic system in radioresistant and radiosensitive mice

**DOI:** 10.1007/s00411-018-0739-4

**Published:** 2018-04-06

**Authors:** Ewa M. Nowosielska, Aneta Cheda, Robert Zdanowski, Sławomir Lewicki, Bobby R. Scott, Marek K. Janiak

**Affiliations:** 1 Department of Radiobiology and Radiation Protection, Military Institute of Hygiene and Epidemiology, 4 Kozielska St., 01-163 Warsaw, Poland; 2 Department of Regenerative Medicine and Cell Biology, Military Institute of Hygiene and Epidemiology, 4 Kozielska St., 01-163 Warsaw, Poland; 3 Lovelace Respiratory Research Institute, 2425 Ridgecrest Drive SE, Albuquerque, 87108 NM USA

**Keywords:** Low-level radiation, Tritiated water, Artificial tumour colonies, NK cells, Macrophages, Cytokines

## Abstract

Tritium is a potentially significant source of internal radiation exposure which, at high levels, can be carcinogenic. We evaluated whether single intraperitoneal injection of BALB/c and C57BL/6 mice with tritiated water (HTO) leading to exposure to low (0.01 or 0.1 Gy) and intermediate (1.0 Gy) cumulative whole-body doses of β radiation is immunosuppressive, as judged by enhancement of artificial tumour metastases, functioning of NK lymphocytes and macrophages, circulating cytokine’s levels, and numbers of bone marrow, spleen, and peripheral blood cells. We demonstrate that internal contamination of radiosensitive BALB/c and radioresistant C57BL/6 mice with HTO at all the absorbed doses tested did not affect the development of neoplastic colonies in the lungs caused by intravenous injection of syngeneic cancer cells. However, internal exposure of BALB/c and C57BL/6 mice to 0.1 and 0.01 Gy of β radiation, respectively, up-regulated cytotoxic activity of and IFN-γ synthesis in NK lymphocytes and boosted macrophage secretion of nitric oxide. Internal contamination with HTO did not affect the serum levels of pro- (IL-1β, IL-2, IL-6, TNF-α,) and anti-inflammatory (IL-1Ra, IL-4, IL-10) cytokines. In addition, exposure of mice of both strains to low and intermediate doses from the tritium-emitted β-particles did not result in any significant changes in the numbers of bone marrow, spleen, and peripheral blood cells. Overall, our data indicate that internal tritium contamination of both radiosensitive and radioresistant mice leading to low and intermediate absorbed β-radiation doses is not immunosuppressive but may enhance some but not all components of anticancer immunity.

## Introduction

In contrast to the well-characterized effects of external radiation exposures, biological effects of internal contamination with radioisotopes are not as well characterized and understood, especially for low and intermediate cumulative radiation doses. Hence, it was recommended that a review be conducted of the relevant risk of internal radioisotope exposures (COMARE 2004). One of the significant sources of internal radiation exposure of workers and members of the public is tritium (^3^H), a β-emitting isotope of hydrogen with low-to-intermediate values of linear energy transfer (LET). Naturally-occurring tritium is extremely rare, but it is a common by-product of nuclear reactors and is also used by a number of industries as well as for research and diagnostic purposes (UNSCEAR 2008 2010). ^3^H binds with hydroxyl radicals to form tritiated water (HTO) that is easily internalized and distributed throughout the organism. Hence, internal contamination with tritium leads to the rather uniform irradiation of the body.

According to the suggestion by the Canadian delegation to United Nations Scientific Committee on the Effects of Atomic Radiation (UNSCEAR 2010), potential adverse biological effects of tritium should be of special interest because there is evidence that its relative biological effectiveness (RBE) factor may be as high as two or more (reviewed in Little and Lambert 2008; Straume and Carsten 1993) and the regulatory limits on releases of ^3^H in the environment appear to be relatively large. Moreover, in view of the growing contribution of the atomic energy production to the world inventory of tritium, interest should be focused on possible adverse and/or beneficial biological effects of exposures to this radioisotope.

According to the linear no-threshold hypothesis, even low-level whole-body exposures to low-LET radiation will lead to an increased risk of cancer development (UNSCEAR 2000). However, the LNT model is no longer considered credible as the model implies that the same impacts of high-radiation doses, which are immunosuppressive, occur at low doses, which have been demonstrated by our group to be immune system enhancing. Initiation and progression of a malignant neoplasm depend on the composition and function of the specific tumour niche (Barcellos-Hoff 2007), where an important role is played by cells of the immune system that generate both anti- and pro-neoplastic as well as anti- and pro-inflammatory responses (Demaria et al. 2010; DeVisser et al. 2006; Janiak et al. 2017). Diverse leukocyte populations found within growing tumours have been shown to adopt various phenotypes that can differentially affect tumour progression (Lewis and Pollard 2006; Mantovani et al. 2008). Most of these cells belong to the innate immune system (DeVisser et al. 2006). One of the recently recognized important functions of this system is triggering and/or sustaining inflammation (Balkwill et al. 2005) which promotes tumour growth, invasion, and metastases (Mantovani et al. 2008; Zeh and Lotze 2005). Among cells which readily localize to sites of inflammation are monocytes which regulate local inflammatory responses (Balkwill et al. 2005; Ibuki and Goto 2004; Lotze and Tracey 2005; Mills et al. 2000; Rubartelli and Lotze 2007; Zeh and Lotze 2005) and natural killer (NK) lymphocytes (Degli-Esposti and Smyth 2005; Empson et al. 2010; Hamerman et al. 2005) which, depending on the context, produce either pro- (IFN-γ, TNF-α) or anti-inflammatory (TGF-β, IL-10) cytokines (Cooper et al. 2001; Cuturi et al. 1989; Dalbeth et al. 2004; Grant et al. 2008). Both activated macrophages (Mφ) and NK lymphocytes have long been recognized as the first-line cytotoxic effectors aimed at neoplastic cells (Empson et al. 2010; Lanier 2008). Importantly, it was repeatedly demonstrated by our group and other researchers that low-level whole-body exposures of mice and rats to low-LET ionizing radiation can result in suppression of both primary and secondary neoplasms and that the effect coexists with up-regulated cytolytic function of NK lymphocytes and Mφ, accompanied by the enhanced secretion by these cells of pro-inflammatory cytokines (Cai 1999; Cheda et al. 2004b, 2008; Hashimoto et al. 1999; Ishii et al. 1996; Janiak et al. 2006, Nowosielska at al. 2006b, 2010, 2011). On the other hand, exposure to ionizing radiation may also activate pro-invasive and pro-metastatic activities of the immune cells associated with inflammation in the tumour site (reviewed in Madani et al. 2008). Indeed, one of the manifestations of the inflammatory microenvironment is suppression of anti-tumour immunity (Demaria et al. 2010). Although it was shown that a high-dose (4 Gy) irradiation promotes carcinogenesis by inducing a ‘hospitable’ tissue environment (Barcellos-Hoff et al. 2005; Barcellos-Hoff and Nguyen 2009), it is not clear whether a similar effect can be instigated by low-level exposures to low-LET radiation and/or if the outcomes of low-level exposures are qualitatively different from those of the higher dose exposures.

The concept of the present investigation was based on the assumptions that: (a) health risks from internal contamination with tritium (mostly in the form of HTO) are possibly underestimated, (b) the most significant late-occurring health effect of such a contamination is cancer whose development is controlled by anti-tumour immune cells which can also actively up-regulate the tumour-promoting inflammation, and (c) the effects of internal incorporation of HTO on the immune and inflammatory responses related to malignancy are unknown. Hence, in the research discussed in the present paper we evaluated whether internal contamination with HTO of mice from two strains with different radiosensitivities and immune phenotypes can diminish the immune system functioning and thereby enhance the development of pulmonary tumour metastases and whether this effect can be linked to alterations in the functions of NK lymphocytes and Mφ, production of pro- and anti-inflammatory cytokines, and/or the haematopoietic system. Thus, the focus of this paper was on possible suppression of anticancer immunity by internal tritium contamination rather than on cancer induction by the contamination.

## Materials and methods

### Animals

6-to-8-week-old male BALB/c mice (the relatively radiosensitive Th2-type responders with dominating M2-type Mφ) obtained from the Nofer Institute of Occupational Medicine, Lodz, Poland, and C57BL/6 mice (the relatively radioresistant strain biased toward the Th1-type lymphocyte- and M1-type Mφ-mediated responses) obtained from the Mossakowski Medical Research Centre, Polish Academy of Sciences, Warsaw, Poland, were used for the experiments. The mice were divided into four experimental groups: three groups of the animals contaminated with HTO at three different exposure levels and one group of the control, uncontaminated mice.

All the mice were maintained under specific pathogen-free conditions. During the experiments, the animals were provided with a natural daily cycle (12-h photoperiod), had access to food and water ad libitum and were housed in a Modular Animal Caging System^®^—MACS Mobile Units (Alternative Design, Siloam Springs, USA). The living conditions and health of the mice were regularly monitored by a veterinarian. The investigations were carried out by permission of the Local Ethical Committee for Experimentation on Animals at the National Medicines Institute in Warsaw.

**Fig. 1:**
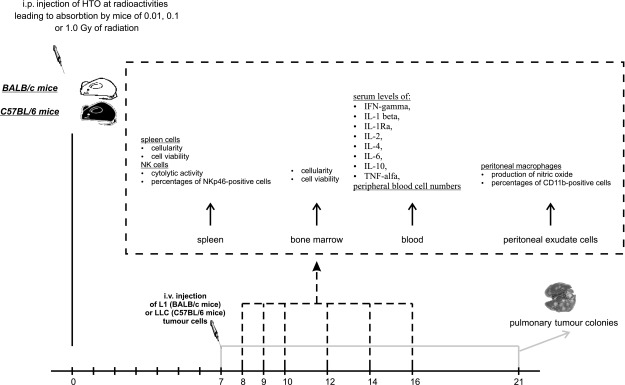
Outline of the experimental procedures. For the assessment of the selected parameters in cells and the serum obtained from the spleen, bone marrow, peritoneal exudates and peripheral blood on the six selected days post-injection of HTO, we used 8 mice per group per day (i.e., 48 mice per group). The experiments were repeated twice, so the total number of animals per group equalled to 96. For the quantification of the neoplastic colonies in the lungs, 24 mice per group were used; the experiments were repeated twice, so the total number of the animals per group was 48

All the experimental procedures described below are outlined in Fig. 1.

### Internal contamination with tritium

The animals were intraperitoneally (i.p.) injected with tritiated water (HTO, PerkinElmer Shared Services sp. z.o.o., Cracow, Poland) at the concentrations of tritium of 0.888, 8.88, or 37 GBq/L (the respective radioactivities equalled to 0.888, 8.88, or 88.8 MBq) so that the calculated total absorbed doses of radiation were 0.01, 0.1, or 1.0 Gy per mouse, respectively. The absorbed doses for the total body were calculated according to the formula described by Tsuchiya et al. (1988):

$${d_\beta }={E_{\beta ~}}\, \cdot \,Q\, \cdot \,k$$

where *d*_*β*_ is the absorbed dose rate [Gy/day], *E*_*β*_ is the average energy of β-rays, *Q* is the radioactivity concentration in tissues [µCi/g], and *k* is a conversion coefficient which has uncertainty that could not be addresses here. The total absorbed doses were calculated taking into account the effective biological half-life of HTO of 2.3 days (Umata et al. 2009). From day 7 post-injection of HTO, i.e., when much of the injected radioactivity was naturally removed from the body or had been lost due to physical decay, blood, spleen, bone marrow, and peritoneal exudate samples were collected on the 8th, 9th, 10th, 12th, 14th, and 16th day after the injection of HTO.

### Tumour cells

L1 sarcoma cells (L1, syngeneic for BALB/c mice) were obtained from the Maria Skłodowska-Curie Memorial Cancer Centre and Institute of Oncology, Warsaw, Poland, and Lewis Lung Carcinoma cells (LLC, syngeneic for C57BL/6 mice) were obtained from the Polish Academy of Sciences Ludwik Hirszfeld Institute of Immunology and Experimental Therapy, Wroclaw, Poland, and used for producing neoplastic colonies (artificial metastases) in the lungs of BALB/c and C57BL/6 mice, respectively. YAC-1 lymphoma cells were obtained from the Polish Academy of Sciences Ludwik Hirszfeld Institute of Immunology and Experimental Therapy, Wroclaw, Poland, and used as targets in the NK cell-mediated cytotoxicity assays. The cells were maintained in a culture medium (CM) composed of the RPMI-1640 medium with L-glutamine (PAN BIOTECH, IMMUNIQ, Zory, Poland), 10% FBS (PAN BIOTECH, IMMUNIQ, Zory, Poland), 100 U/ml penicillin (Polfa, Warsaw, Poland) and 100 mg/ml streptomycin (Polfa, Warsaw, Poland) in standard conditions (SC): humidified atmosphere of 95% air and 5% CO_2_ at 37 °C.

### Tumour colony assay

Fourteen days after the subcutaneous (s.c.) transplantation of 10^6^ L1 or LLC cells to three BALB/c or C57BL/6 mice, respectively, the developed tumours were removed, minced, and incubated for 30 min at room temperature (RT) in 0.25% trypsin-EDTA (Gibco, Warsaw, Poland) and standard DNase I enzyme solution (Sigma, Poznan, Poland). After that, the cells were washed and resuspended in CM. For the assay, 7 days after the injection of HTO 2.5 × 10^5^ L1 or LLC cells per mouse were intravenously (i.v.) injected (24 mice were used per group). Fourteen days later (i.e., on the 21st day post-injection of HTO) the animals were euthanized and total numbers of macroscopic colonies were counted on the surface of the dissected lungs (Cheda et al. 2004b; Nowosielska et al. 2006b).

### Preparation of the NK cell-enriched splenocytes

The procedure was described previously (Cheda et al. 2004b). Briefly, on the 8th, 9th, 10th, 12th, 14th, and 16th day post-injection of HTO, the mice were euthanized and single-cell suspensions prepared from the spleens were suspended in CM and incubated on glass Petri dishes for 40 min in SC; on each day the cells were collected and pooled from eight mice. After the incubation, non-adherent cells were collected and erythrocytes were lysed. The remaining cells were then washed, resuspended in CM and passed through a nylon wool column to obtain the wool-non-adherent NK cell-enriched splenocytes (NK cells) containing approx. 12% of the NK-type lymphocytes, as estimated by labelling with the anti-mouse Pan-NK Cells DX5 antibody (Becton Dickinson, Warsaw, Poland).

### Preparation of peritoneal Mφ

The procedure was described previously (Nowosielska et al. 2006b). Briefly, three days before the collection of peritoneal exudate cells mice were i.p. injected with 1 ml of 10% Sephadex G-25 (Pharmacia, Uppsala, Sweden) and peritoneal exudate was collected from euthanized mice on the 8th, 9th, 10th, 12th, 14th, and 16th day post-injection of HTO; on each day the cells were collected and pooled from eight mice. The cells were resuspended in CM, and incubated on glass Petri dishes for 2 h in SC. The glass-adherent cells containing approx. 80% cells with morphological features of a typical macrophage were then harvested and resuspended in CM.

### NK cell-mediated cytotoxicity assay

Cytolytic activity of the NK cells was measured using the ^51^Cr-release assay (Cheda et al. 2004b). Briefly, the YAC-1 target (T) cells (10^6^ in 100 µl CM) were incubated in SC for 1.5 h with 5.55 MBq of sodium chromate (Na_2_^51^CrO_4_; Polatom, Otwock-Swierk, Poland). Then, the cells were washed with PBS (BioMed-LUBLIN, Lublin, Poland) and added to the effector (E) NK cells at 100:1 E:T cell ratio; for each experimental group five samples were used. After the 4-h incubation in SC, aliquots of the cell-free supernatants were harvested and the radioactivity of ^51^Cr released from T was measured in a γ-counter (Auto-Gamma Cobra II; Canberra-Packard, Warsaw, Poland). The ratio of the NK cell-mediated cytolytic activity was calculated using the formula:

$$\% \;{\text{cytotoxicity}}=\frac{{{\text{experimental}}\;{\text{release}}\;{\text{of}}\;{\text{Cr}} - 51-{\text{spontaneous}}\;{\text{release}}\;{\text{of}}\;{\text{Cr}} - 51}}{{{\text{maximum}}\;{\text{release}}\;{\text{of}}\;{\text{Cr}} - 51-{\text{spontaneous}}\;{\text{release}}\;{\text{of}}\;{\text{Cr}} - 51}} \times 100$$

### Production of nitric oxide (NO) by Mφ

Nitric oxide (NO) synthesized by activated Mφ was quantitated by measuring the level of the nitrite ion (NO_2_^−^) in the incubation medium (Nowosielska et al. 2011). Mφ were suspended in CM supplemented with 50 U/ml interferon-γ (IFN-γ; Sigma, Poznan, Poland) and 100 ng/ml lipopolysaccharide (LPS; Sigma) and incubated for 48 h in SC; for each experimental group eight samples were used. After that, 100 µl of the supernatant was mixed with 100 µl of the Griess reagent and kept in the dark for 10 min at RT. Absorbance at 540 nm was then measured using the microplate spectrophotometer Epoch™ (BioTek^®^ Instruments, Inc., Vermont, USA). The obtained data were analyzed with use of the reader software Gen5™ 2.0 (BioTek^®^ Instruments, Inc., Vermont, USA).

### Spleen and bone marrow cellularity

On the 8th, 9th, 10th, 12th, 14th, and 16th day after injection of HTO spleens were removed from the anesthetized mice, minced, and the obtained cells were suspended in PBS (BioMed-LUBLIN, Lublin, Poland). On the same days, post-injection samples of bone marrow were collected from the femurs of the anesthetized mice and suspended in PBS (BioMed-LUBLIN). On each day the spleens and bone marrow were collected and pooled from eight mice. The resulting single-cell suspensions were quantitated in a mammalian cell counter NucleoCounter^®^ NC-100™ (ChemoMetec, Allerød, Denmark).

### Peripheral blood cell counts

On the 8th, 9th, 10th, 12th, 14th, and 16th day post-injection of HTO blood samples were collected by heart punctures of the anesthetized mice; on each day the blood was collected from eight mice. Blood cell counts were estimated in a haematological analyser Mythic 18 (Cormay, Lomianki, Poland).

### Percentages of NK cells and Mφ

NK cell-enriched splenocytes and peritoneal macrophage-enriched cell suspensions were incubated with the anti-mouse CD335 (NKp46) FITC and anti-mouse CD11b APC antibodies, respectively, and five samples from each experimental group were analyzed in a flow cytometer to estimate the percentage of the NK cells and Mφ in the cell suspensions under study.

### Production of cytokines

On the 8th, 9th, 10th, 12th, 14th, and 16th day after the injection of HTO serum samples were prepared from peripheral blood obtained by heart punctures of the anesthetized mice; on each day the blood was collected and pooled from eight mice. The obtained serum samples were frozen at − 70 °C and then, after defrosting, assayed for the levels of:

- IL-2, IL-4, IL-6, IL-10, IFN-γ, and TNF-α, using the Mouse Th1/Th2/Th17 CBA Kits (Becton Dickinson, Warsaw, Poland)—with use of flow cytometry;
- IL-1β and IL-1Ra, using the respective Quantikine^®^ ELISA Mouse kits (R&D Systems, Inc., Minneapolis, USA)—with use of ELISA method employing the Epoch™ microplate spectrophotometer (BioTek^®^ Instruments) and the ELx50™ microplate strip washer (BioTek^®^ Instruments).

### Statistical analysis

Inter-group differences in the cytotoxic function of NK cells, spleen and bone marrow cellularities, peripheral blood cell counts, or the serum levels of cytokines were analyzed using the Mann–Whitney *U* test for non-parametric trials with *p* values less than 0.05 regarded as significant.

## Results

**Fig. 2:**
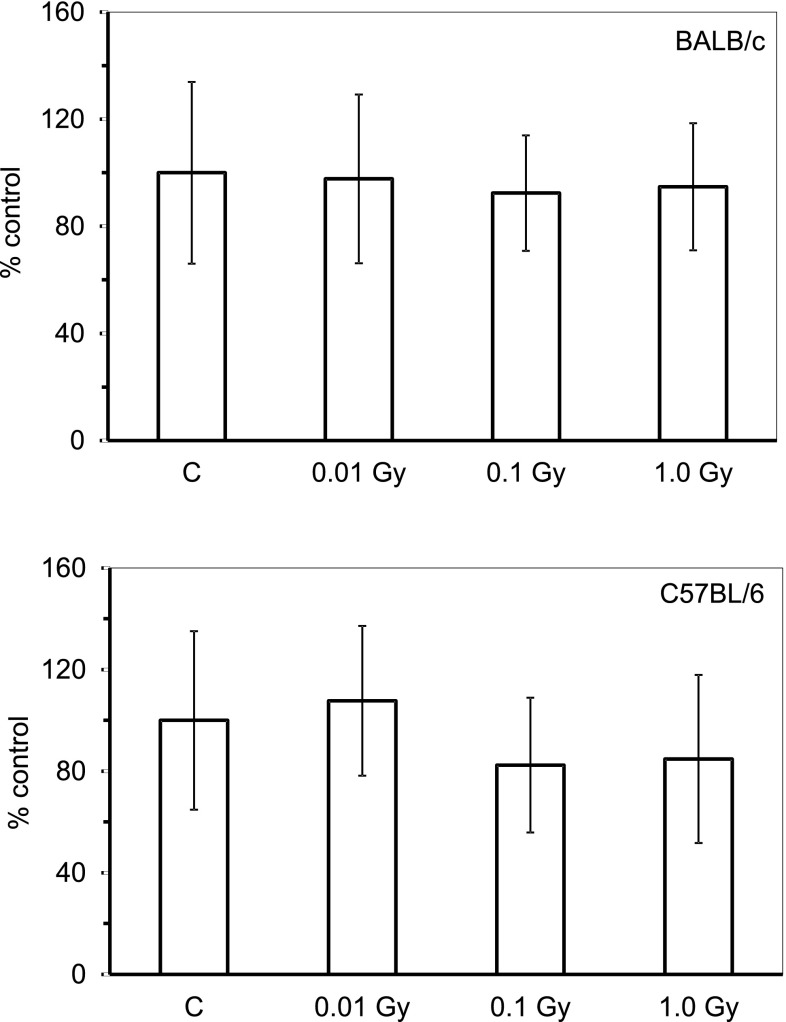
Relative numbers [% of the control value] of the artificial tumour colonies in the lungs of BALB/c or C57BL/6 mice 21 days after injection of HTO. Mean values obtained from two experiments each using 24 animals per group (i.e., a total of 48 animals in each group) are presented. *BALB/c* the relatively radiosensitive mice, *C57BL/6* the relatively radioresistant mice, *C* control mice, uninjected with HTO, *0.01 Gy* mice injected with HTO so that the total absorbed dose of radiation was 0.01 Gy per mouse, *0.1 Gy* mice injected with HTO so that the total absorbed dose of radiation was 0.1 Gy per mouse, *1.0 Gy* mice injected with HTO so that the total absorbed dose of radiation was 1.0 Gy per mouse

As shown in Fig. 2, contamination with HTO leading to calculated whole-body doses of 0.01, 0.1 and 1.0 Gy was not associated with any significant changes in the number of the developed syngeneic tumour colonies in the lungs of both BALB/c and C57BL/6 mice (expressed as percentages of the average control values obtained in the sham-exposed animals) compared to the control, uncontaminated animals in which the respective numbers of pulmonary tumour colonies varied between 15 and 20 per mouse (data not shown).

**Fig. 3:**
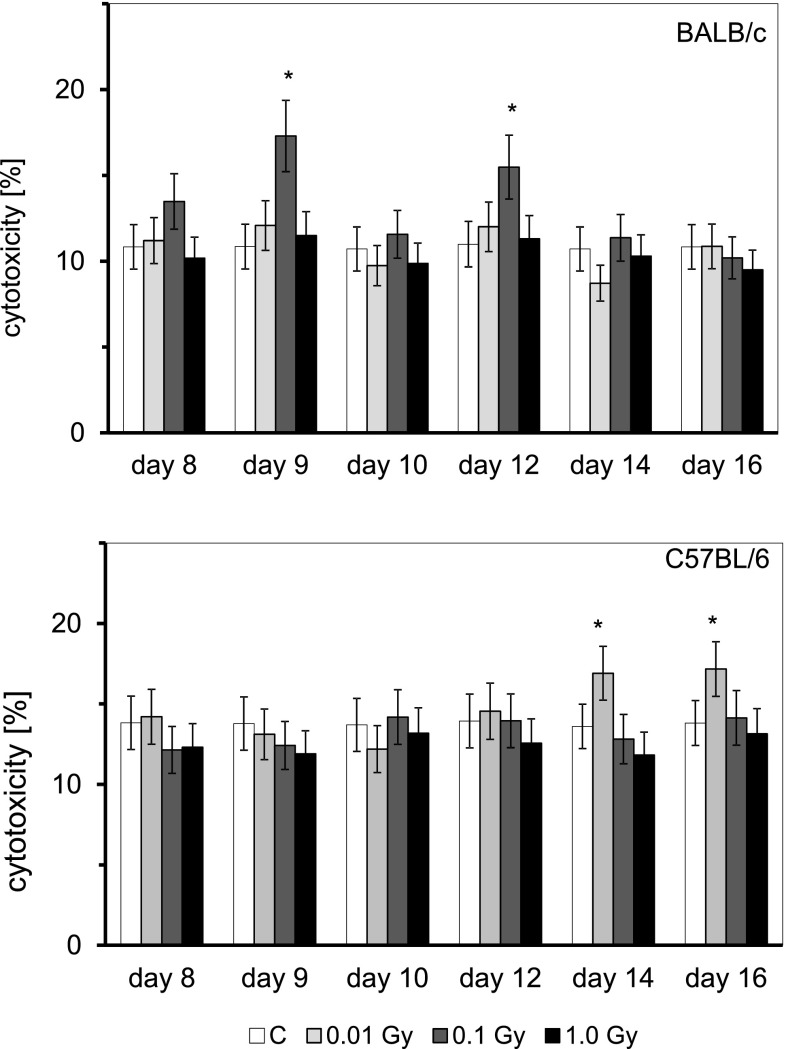
Cytotoxic activity [%] of NK cells obtained from BALB/c or C57BL/6 mice on various days after contamination with HTO. Mean values obtained from two experiments each using 8 animals per group per day (i.e., a total of 16 animals in each group per day) are presented. *BALB/c* the relatively radiosensitive mice, *C57BL/6* the relatively radioresistant mice, *C* control mice, uninjected with HTO, *0.01 Gy* mice injected with HTO so that the total absorbed dose of radiation was 0.01 Gy per mouse, *0.1 Gy* mice injected with HTO so that the total absorbed dose of radiation was 0.1 Gy per mouse, *1.0 Gy* mice injected with HTO so that the total absorbed dose of radiation was 1.0 Gy per mouse, *day 8 … day 16* days after injection of HTO. *Statistically significant (*p* < 0.05) difference from the results obtained in the control mice

In two preliminary experiments conducted on BALB/c and C57BL/6 mice exposed to 0.1 Gy of radiation, no significant differences in the cytotoxic activity of the NK cell-enriched splenocytes were detected between the control and irradiated groups beyond days 14–16 (and up to day 22) after the injection of HTO. Hence, in the following assays this activity was estimated only until day 16 post-injection. As shown in Fig. 3, contamination of BALB/c mice with HTO led to the significant enhancement of the cytolytic function of the NK cells only in the group of the animals exposed to β-radiation at the absorbed dose of 0.1 Gy and only on the 9th and 12th days post-injection of HTO. In contrast, contamination with HTO of C57BL/6 mice resulted in the significant stimulation of the NK cell-mediated cytotoxicity only in the group of the animals with an absorbed dose of 0.01 Gy of radiation and only on the 14th and 16th days after the application of HTO. Cytometric analysis of the anti-mouse CD335 (NKp46)-labelled splenic NK cells obtained from contaminated BALB/c and C57BL/6 mice showed that for each absorbed dose studied, there was no significant increase in the percentages of these cells; this effect was comparable in the two strains of mice and was detectable both before and after the splenocyte suspension was purified on the nylon wool columns (Table 1).

**Table 1:** Percentages of CD335 (NKp46)-positive splenocytes obtained from BALB/c or C57BL/6 mice contaminated with HTO

	C		0.01 Gy		0.1 Gy		1.0 Gy	
	No passage	After passage	No passage	After passage	No passage	After passage	No passage	After passage
BALB/c								
Day 8	11.7 ± 1.4	17.6 ± 2.2	13.7 ± 1.8	20.6 ± 2.8	13.5 ± 1.8	20.2 ± 2.7	13.7 ± 1.8	19.4 ± 2.6
Day 9			13.5 ± 1.8	20.4 ± 2.7	13.2 ± 1.8	20.5 ± 2.8	13.0 ± 1.7	20.4 ± 2.8
Day 10			14.1 ± 1.9	20.1 ± 2.7	13.8 ± 1.9	20.1 ± 2.7	13.2 ± 1.8	20.1 ± 2.7
Day 12			12.9 ± 1.7	20.5 ± 2.8	13.1 ± 1.8	19.9 ± 2.7	12.7 ± 1.7	21.4 ± 2.9
Day 14			13.3 ± 1.8	20.9 ± 2.8	13.7 ± 1.8	20.6 ± 2.8	12.9 ± 1.7	19.9 ± 2.7
Day 16			13.9 ± 1.9	20.3 ± 2.7	13.5 ± 1.8	20.7 ± 2.8	13.2 ± 1.8	20.6 ± 2.8
C57BL/6								
Day 8	6.4 ± 0.8	8.5 ± 1.0	7.2 ± 1.0	10.5 ± 1.4	7.4 ± 1.0	10.6 ± 1.4	6.7 ± 0.9	10.1 ± 1.4
Day 9			7.6 ± 1.0	10.2 ± 1.4	7.7 ± 1.0	10.4 ± 1.4	7.1 ± 1.0	9.5 ± 1.3
Day 10			7.1 ± 1.0	10.6 ± 1.4	7.3 ± 1.0	10.8 ± 1.5	6.6 ± 0.9	9.8 ± 1.3
Day 12			7.7 ± 1.0	10.7 ± 1.4	7.8 ± 1.1	10.9 ± 1.5	7.2 ± 1.0	10.0 ± 1.3
Day 14			7.1 ± 1.0	10.4 ± 1.4	7.3 ± 1.0	10.5 ± 1.4	6.6 ± 0.9	9.6 ± 1.3
Day 16			7.4 ± 1.0	10.5 ± 1.4	7.5 ± 1.0	10.6 ± 1.4	6.9 ± 0.9	9.7 ± 1.3

Mean values ± SD obtained from two experiments conducted on 8 animals per group per day (total number of animals in each group per day equaled to 16) are presented. The control values are means from days 8,9,10,12,14,16

*BALB/c* relatively radiosensitive mice, *C57BL/6* relatively radioresistant mice, *C* control mice, not contaminated with HTO, *0.01 Gy* mice contaminated with HTO so that the total absorbed dose of radiation was 0.01 Gy per mouse, *0.1 Gy* mice contaminated with HTO so that the total absorbed dose of radiation was 0.1 Gy per mouse, *1.0 Gy* mice contaminated with HTO so that the total absorbed dose of radiation was 1.0 Gy per mouse, *Day 8 … Day 16* days after contamination with HTO, *no passage* single-cell suspensions prepared from the spleens, *after passage* single-cell suspensions prepared from the spleens and passed through a nylon wool column

**Fig. 4:**
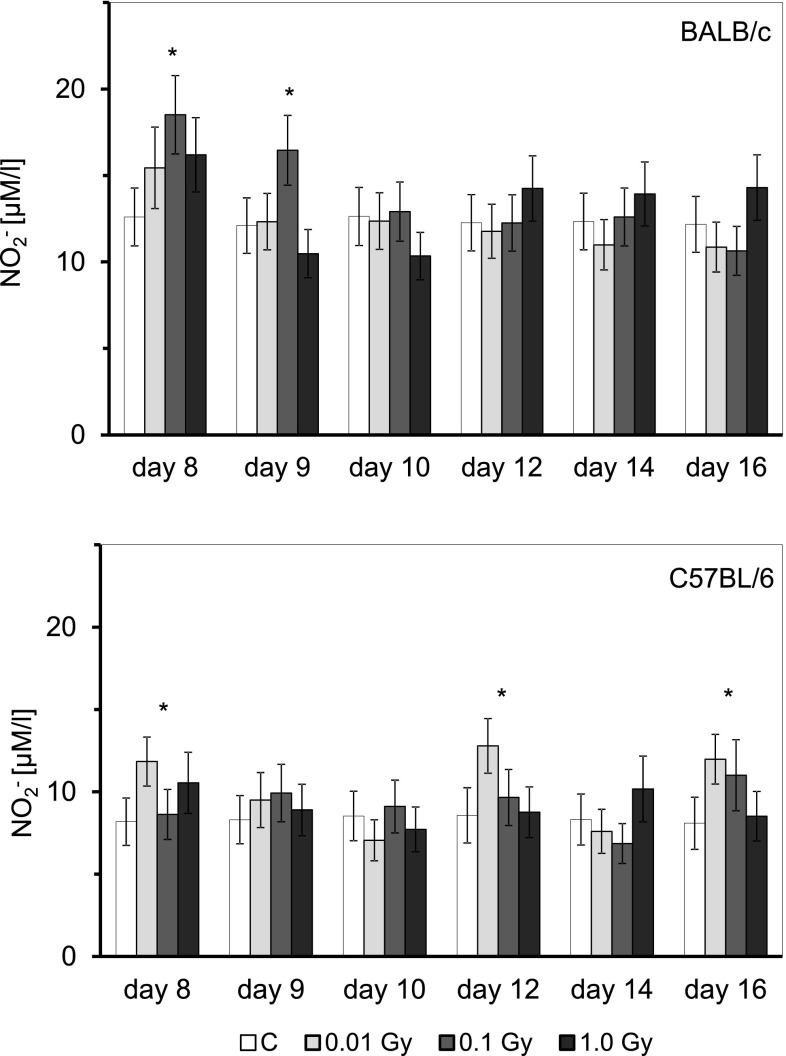
Production of NO [µM/L] by peritoneal Mφ obtained from BALB/c or C57BL/6 mice on various days after contamination with HTO. Mean values obtained from two experiments each using 8 animals per group per day (i.e., a total of 16 animals in each group per day) are presented. *BALB/c* the relatively radiosensitive mice, *C57BL/6* the relatively radioresistant mice, *C* control mice, uninjected with HTO, *0.01 Gy* mice injected with HTO so that the total absorbed dose of radiation was 0.01 Gy per mouse, *0.1 Gy* mice injected with HTO so that the total absorbed dose of radiation was 0.1 Gy per mouse, *1.0 Gy* mice injected with HTO so that the total absorbed dose of radiation was 1.0 Gy per mouse, *day 8 … day 16* days after injection of HTO. *Statistically significant (*p* < 0.05) difference from the results obtained in the control mice

As indicated in Fig. 4, activated Mφ collected from BALB/c and C57BL/6 mice contaminated with HTO produced more nitric oxide than Mφ obtained from uncontaminated animals. However, in BALB/c mice the effect was significantly pronounced between the 8th and 9th day post-injection of HTO when the total absorbed dose equalled to 0.1 Gy, but not 0.01 and 1.0 Gy. In contrast, in C57BL/6 mice the effect was significantly expressed on the 8th, 12th, and 16th days after the HTO injection and only in the animals with an absorbed dose of 0.01 Gy. Cytometric analysis of the anti-mouse CD11b-labelled peritoneal Mφ collected from the HTO-contaminated BALB/c and C57BL/6 mice showed that total absorbed doses of 0.01, 0.1, or 1.0 Gy did not affect the number of these cells obtained from the peritoneal exudates (data not shown).

**Fig. 5:**
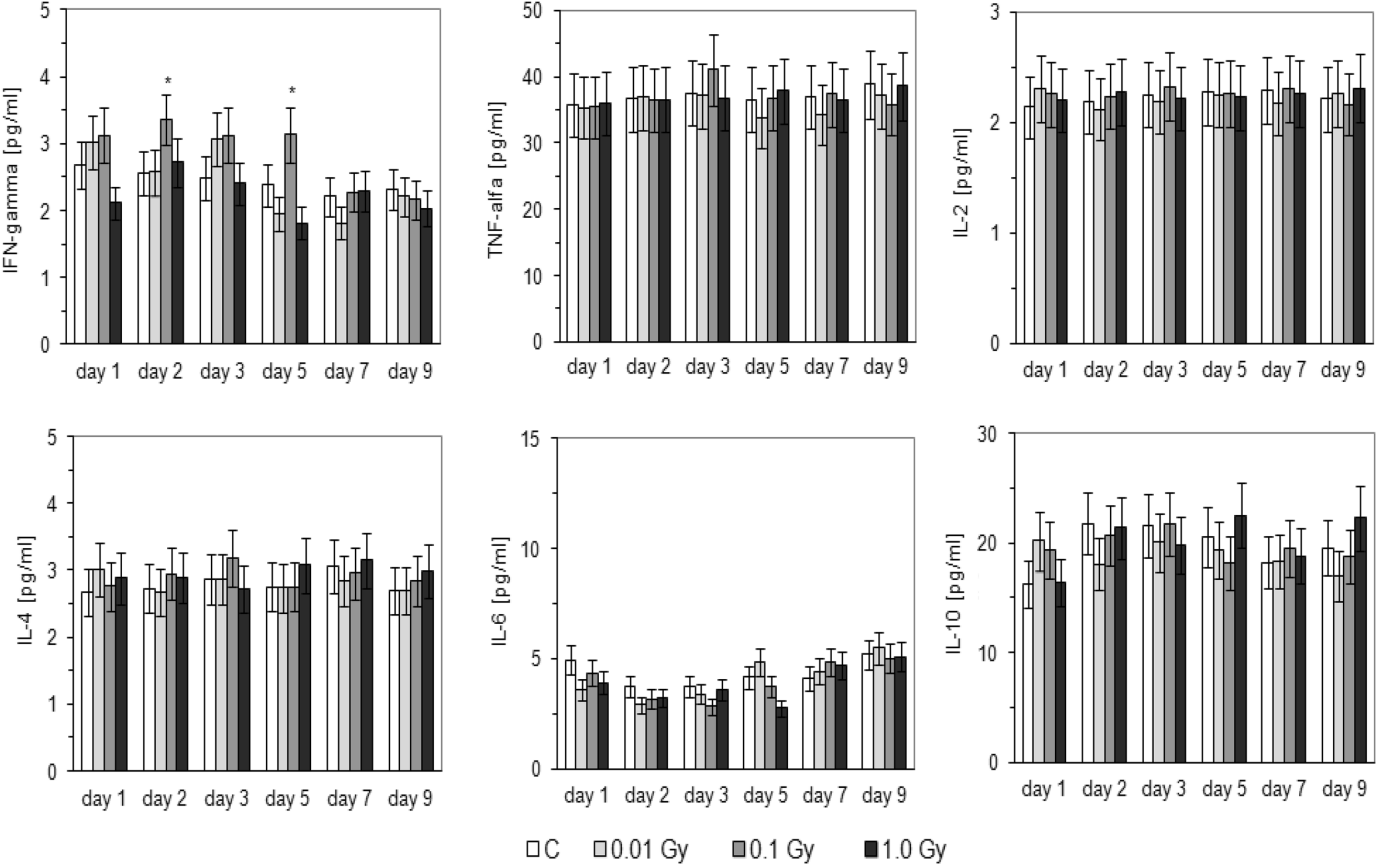
Serum levels [pg/ml] of IFN-γ, TNF-α, IL-2, IL-4, IL-6, IL-10 in BALB/c mice on various days after contamination with HTO. Mean values obtained from two experiments, carried out with use of the CBA kits, each using 8 animals per group per day (i.e., a total of 16 animals in each group per day) are presented. *C* control mice, uninjected with HTO, *0.01 Gy* mice injected with HTO so that the total absorbed dose of radiation was 0.01 Gy per mouse, *0.1 Gy* mice injected with HTO so that the total absorbed dose of radiation was 0.1 Gy per mouse, *1.0 Gy* mice injected with HTO so that the total absorbed dose of radiation was 1.0 Gy per mouse, *day 8 … day 16* days after injection of HTO. *Statistically significant (*p* < 0.05) difference from the results obtained in the control mice

**Fig. 6:**
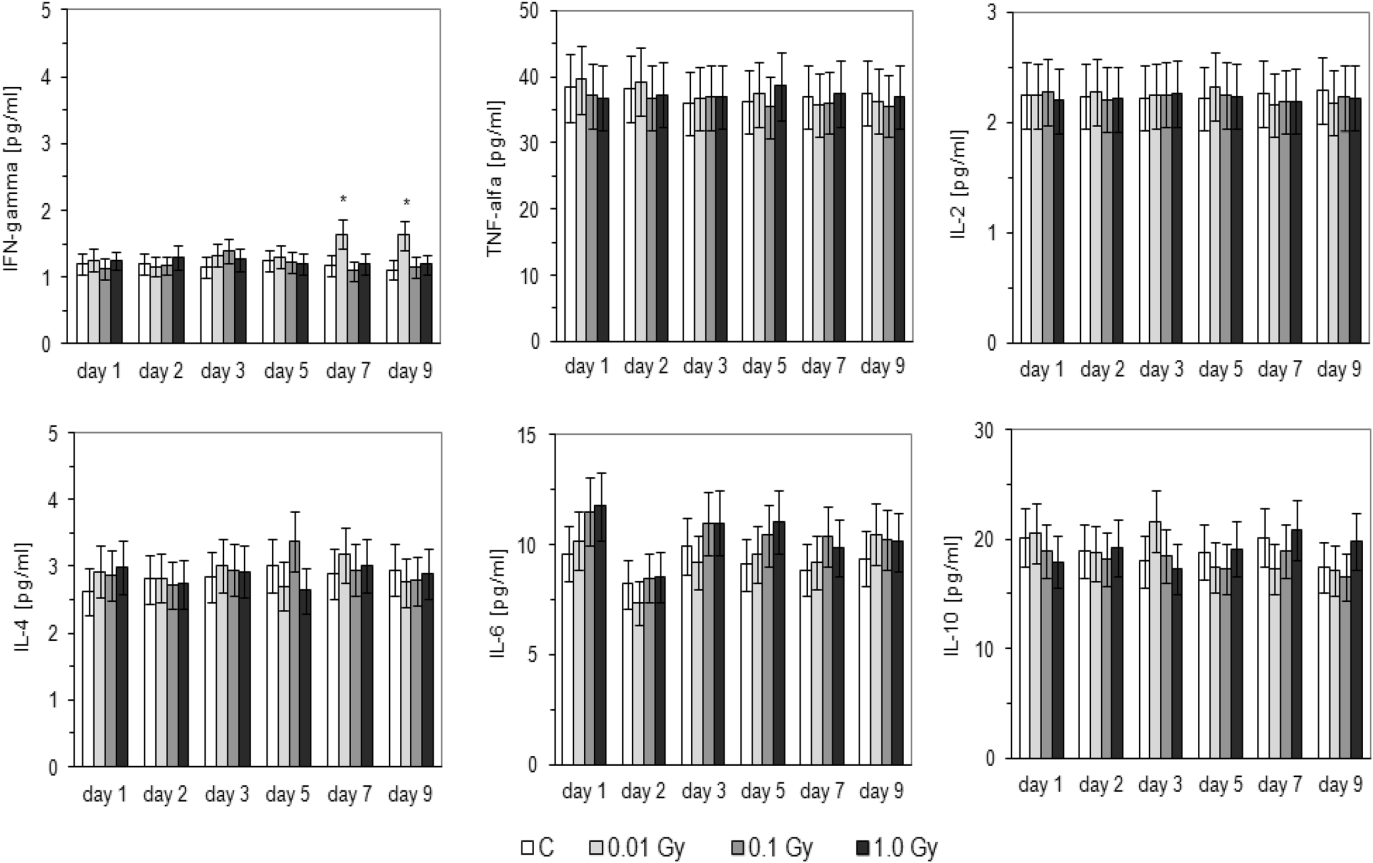
Serum levels [pg/ml] of IFN-γ, TNF-α, IL-2, IL-4, IL-6, IL-10 in C57BL/6 mice on various days after contamination with HTO. Mean values obtained from two experiments, carried out with use of the CBA kits, each using 8 animals per group per day (i.e., a total of 16 animals in each group per day) are presented. *C* control mice, uninjected with HTO, *0.01 Gy* mice injected with HTO so that the total absorbed dose of radiation was 0.01 Gy per mouse, *0.1 Gy* mice injected with HTO so that the total absorbed dose of radiation was 0.1 Gy per mouse, *1.0 Gy* mice injected with HTO so that the total absorbed dose of radiation was 1.0 Gy per mouse, *day 8 … day 16* days after injection of HTO. *Statistically significant (*p* < 0.05) difference from the results obtained in the control mice

As shown in Fig. 5, the serum level of IFN-γ was significantly elevated in the HTO-contaminated BALB/c mice when the total absorbed dose was 0.1 Gy; the effect was pronounced on the 9th and 12th days post-injection of HTO and insignificantly elevated levels of this cytokine were observed also on the 8th and 10th days post-injection. In C57BL/6 mice, the level of IFN-γ was markedly elevated only on the 14th and 16th days after application of HTO when the total absorbed dose was 0.01 Gy (Fig. 6). In contrast to IFN-γ, serum levels of other tested cytokines, i.e., IL-2, IL-4, IL-6, IL-10, and TNF-α, were not significantly affected by contamination of BALB/c (Fig. 5) and C57BL/6 (Fig. 6) mice with HTO.

**Fig. 7:**
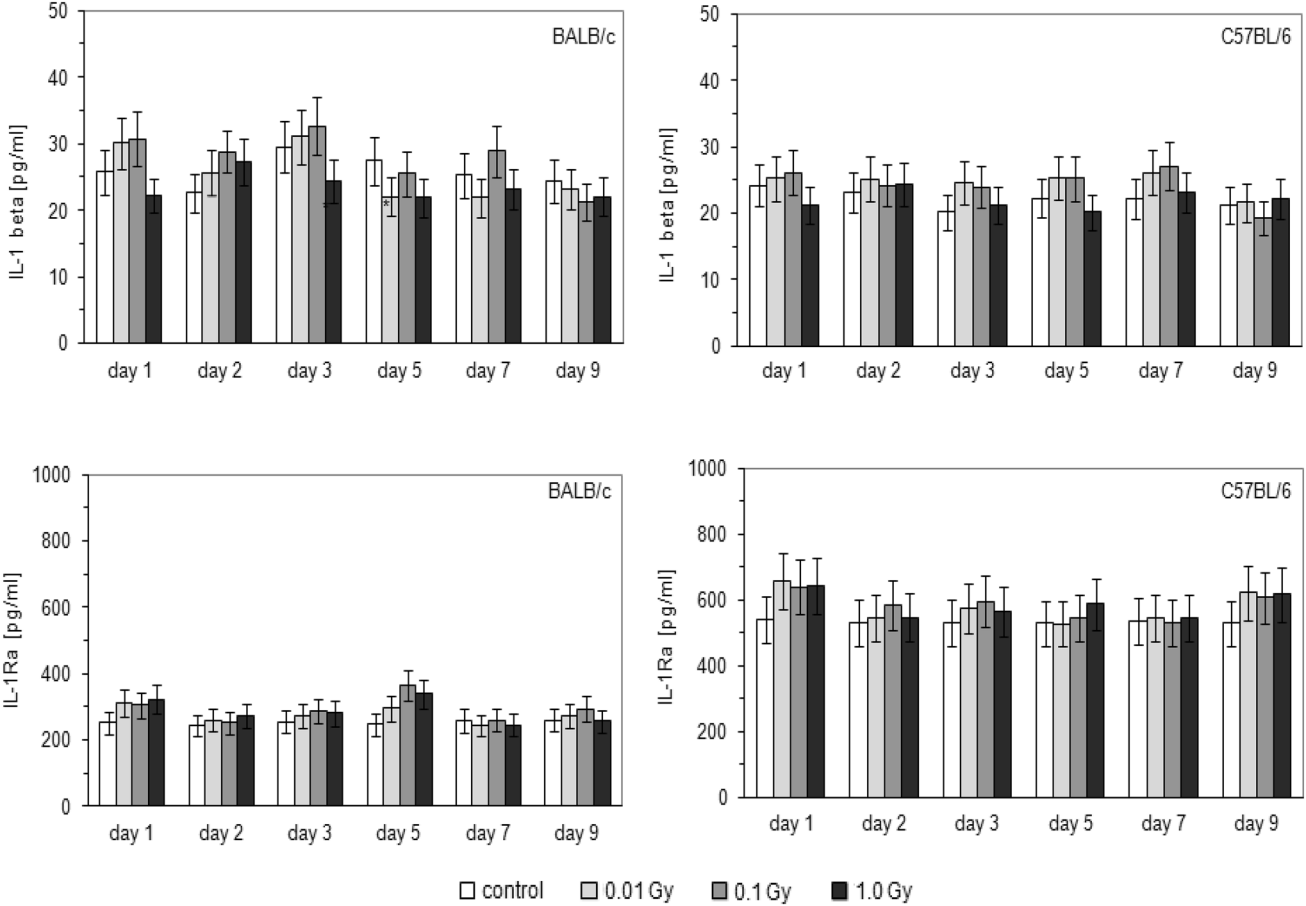
Serum levels [pg/ml] of IL-1β and IL-1Ra in BALB/c and C57BL/6 mice on various days after contamination with HTO. Mean values obtained from two experiments, carried out with use of ELISA method, each using 8 animals per group per day (i.e., a total of 16 animals in each group per day) are presented. *BALB/c* the relatively radiosensitive mice, *C57BL/6* the relatively radioresistant mice, *C* control mice, uninjected with HTO, *0.01 Gy* mice injected with HTO so that the total absorbed dose of radiation was 0.01 Gy per mouse, *0.1 Gy* mice injected with HTO so that the total absorbed dose of radiation was 0.1 Gy per mouse, *1.0 Gy* mice injected with HTO so that the total absorbed dose of radiation was 1.0 Gy per mouse, *day 8 … day 16* days after injection of HTO

As indicated in Fig. 7, insignificant increases in the serum level of IL-1β were detected on the 8th, 9th, and 10th days post-injection of HTO to BALB/c mice when the total absorbed doses were 0.01 and 0.1 Gy; in C57BL/6 mice a similar effect occurred on the 10th, 12th, and 14th days after the injection. Measurements of the serum levels of IL-1Ra (Fig. 7) demonstrated that contamination with HTO leading to absorbed doses of 0.01, 0.1, or 1.0 Gy did not affect the production of this cytokine in BALB/c and C57BL/6 mice, but in the latter animals its levels were approximately twice as high as in the former mice.

No significant differences were detected in the numbers of bone marrow and spleen cells collected from the HTO-contaminated BALB/c and C57BL/6 mice between the 8th and 16th days after the injection of HTO compared to the numbers of these cells collected from the control animals (Table 2). Likewise, contamination with HTO did not lead to any significant changes in the total numbers and viabilities of leukocytes, platelets, and erythrocytes in the peripheral blood collected from the contaminated BALB/c and C57BL/6 mice between the 8th and 16th days post-injection of HTO (data not shown).

**Table 2:** Bone marrow (BM) and spleen (S) cellularities in BALB/c and C57BL/6 mice contaminated with HTO

	C		0.01 Gy		0.1 Gy		1.0 Gy	
	BM (×10^5^)	S (×10^6^)	BM (×10^5^)	S (×10^6^)	BM (×10^5^)	S (×10^6^)	BM (×10^5^)	S (×10^6^)
BALB/c								
Day 8	86.4 ± 24	180.0 ± 50	68.6 ± 18	175.8 ± 42	65.6 ± 17	148.8 ± 49	75.2 ± 21	159.7 ± 45
Day 9			82.5 ± 23	183.6 ± 51	64.5 ± 18	145.2 ± 41	71.8 ± 20	167.2 ± 47
Day 10			89.3 ± 25	184.8 ± 52	74.4 ± 21	206.8 ± 58	82.6 ± 23	173.8 ± 49
Day 12			79.8 ± 22	153.0 ± 43	77.7 ± 22	181.2 ± 51	70.1 ± 20	135.9 ± 38
Day 14			77.6 ± 22	207.0 ± 58	81.6 ± 23	195.6 ± 55	71.5 ± 20	187.6 ± 48
Day 16			76.0 ± 21	223.0 ± 62	86.6 ± 24	140.8 ± 39	81.6 ± 23	171.8 ± 53
C57BL/6								
Day 8	66.4 ± 19	125.3 ± 35	85.7 ± 24	96.6 ± 24	86.5 ± 27	95.2 ± 27	62.3 ± 17	82.2 ± 23
Day 9			72.3 ± 27	95.0 ± 20	61.0 ± 17	90.6 ± 25	62.9 ± 18	86.2 ± 20
Day 10			89.0 ± 27	100.8 ± 25	104.5 ± 29	107.4 ± 30	71.0 ± 24	91.0 ± 23
Day 12			86.6 ± 27	125.8 ± 28	90.6 ± 25	110.8 ± 31	83.8 ± 25	101.9 ± 22
Day 14			95.4 ± 24	106.4 ± 35	83.0 ± 23	114.4 ± 32	79.5 ± 29	99.8 ± 28
Day 16			98.9 ± 28	90.4 ± 25	82.5 ± 23	110.2 ± 31	87.9 ± 25	114.2 ± 32

Mean values ± SD obtained from two experiments conducted on 8 animals per group per day (total number of animals in each group per day equaled to 16) are presented. The control values are means from days 8,9,10,12,14,16

*BALB/c* relatively radiosensitive mice, *C57BL/6* relatively radioresistant mice, *C* control mice, not contaminated with HTO, *0.01 Gy* mice contaminated with HTO so that the total absorbed dose of radiation was 0.01 Gy per mouse, *0.1 Gy* mice contaminated with HTO so that the total absorbed dose of radiation was 0.1 Gy per mouse, *1.0 Gy* mice contaminated with HTO so that the total absorbed dose of radiation was 1.0 Gy per mouse, *Day 8 … Day 16* days after contamination with HTO

## Discussion

The focus of the present study was on identifying possible relations between the internalized tritium contamination and immunosuppression, which could enhance cancer risk. To our knowledge, this is the first attempt to estimate whether single internal contamination with HTO associated with whole-body low (0.01 and 0.1 Gy) and intermediate (1.0 Gy) absorbed doses of β-radiation can enhance the development of artificial neoplastic metastases (the enhancement is a biological marker for immunosuppression) and to record specific immune system responses in mice. For our purposes, we employed one of the routinely used experimental models in which mice are i.p. injected with HTO (Priest et al. 2017; Umata et al. 2009). Such a route of contamination assures fast and relatively uniform distribution of the radioisotope throughout the body and enables a more precise estimation of the absorbed doses of radiation than after ingestion of tritium orally or through inhalation.

The obtained results demonstrate that single internal contamination of both radiosensitive and radioresistant mice with HTO at calculated total absorbed doses of 0.01, 0.1, or 1.0 Gy did not affect the development of the injected cancer cells-related tumour colonies in the lungs (Fig. 2). This observation differs from the results of studies indicating that chronic and/or continuous administration of HTO to mice or rats with much higher absorbed doses (which may be immunosuppressive) than those used in the present investigation can be associated with the development of various neoplasms (reviewed in UNSCEAR 2017). For example, Yamamoto et al. (1995, 1998), who orally administered HTO to female (C57BL/6N x C3H/He) F1 mice from 10 weeks of age at the dose rate to soft tissues of 0.0036–0.24 Gy/day, demonstrated a significant increase in the lifetime incidence of spontaneous non-thymic lymphomas and solid tumours accompanied by shortening of the animals’ lifespan. However, dose rates lower than 0.0036 Gy/day did not produce such effects. Likewise, single or four subsequent (with weekly intervals) i.p. injections of the same mice with HTO at whole-body absorbed doses from 2.0 to 10.5 Gy stimulated the development of solid tumours and malignant T-lymphomas (Seyama et al. 1991). Also, Johnson et al. (1995), who exposed CBA/H mice to HTO at total absorbed doses of 1–3 Gy, showed an increase in the lifetime incidence of myeloid leukaemia from 0.13% in the control group to 6–8% in the exposed animals.

There is no known reason for the radiobiology of tritium to be qualitatively different from that of other low-LET radiation types. Indeed, the spectrum of malignancies detected in laboratory animals following contamination with HTO seems to be similar to that induced by uniform whole-body irradiations with low-LET X- or gamma-rays (Straume and Carsten 1993). In a series of our previous studies we showed that whole-body exposures of BALB/c and C57BL/6 mice to both single and multiple irradiations with X-rays at total doses ranging from 0.05 to 0.2 Gy reproducibly suppressed the development of the injected cancer cell-related neoplastic colonies in the lungs (Cheda et al. 2004a, b, 2005, 2006, 2008, 2009; Janiak et al. 2006; Nowosielska et al. 2005, 2006a, 2008, 2010, 2011, 2012). Since the mice were irradiated before the intravenous inoculation of the syngeneic tumour cells, the low-level exposures to X-rays were thought to stimulate systemic innate anti-neoplastic reactions. Indeed, although we were not able to directly estimate the activities of immune cells in the lungs, a significant stimulation of the cytotoxic functions of the NK cell-enriched splenocytes and the LPS/IFN-γ-stimulated peritoneal macrophages was detected in the X-ray-exposed mice from the two strains. Among the up-regulated functions of these cells were the increased productions of IFN-γ (by the NK-type splenocytes) and NO (by stimulated macrophages). Interestingly, no such activities of NK lymphocytes and macrophages were detected after the exposures of these cells to X-rays in vitro indicating that the radiation-induced up-regulation of the cytolytic functions requires cooperation of the effector cells with components of the in vivo environment which are absent in the in vitro cultures (Cheda et al. 2004a, b, 2005, 2006, 2008, 2009; Janiak et al. 2006; Nowosielska et al. 2005, 2006a, 2008, 2010, 2011, 2012).

As demonstrated in the current study, internal contamination of BALB/c and C57BL/6 mice with HTO at calculated total absorbed doses of 0.1 and 0.01 Gy, respectively, stimulated activated peritoneal macrophages to synthesize NO (Fig. 3)—a molecule responsible for cytotoxic activity of these cells (Nowosielska et al. 2011). Moreover, the same total absorbed doses of the HTO-derived radiation enhanced cytolytic activity of NK splenocytes (Fig. 4) in both the radiosensitive BALB/c and the radioresistant C57BL/6 mice. This effect was accompanied by the increased serum levels of IFN-γ in the respective groups of mice (Figs. 5, 6). Notably, the kinetics of the enhanced production of this cytokine was similar to the directly cytolytic function of NK cells towards susceptible tumour cells (Figs. 4, 5, 6). However, despite the HTO-boosted cytotoxic activities of NK lymphocytes and macrophages, in the present investigations no differences in the numbers of the artificial pulmonary metastases were detected between the contaminated and uncontaminated animals. This lack of a beneficial immunological effect against artificial metastases may be, at least partially, explained by the observation that internal exposures of BALB/c and C57BL/6 mice to both low (0.01 and 0.1 Gy) and intermediate (1.0 Gy) absorbed doses of the tritium-emitted radiation did not affect the systemic production of a number of pro- (IL-1β, IL-2, IL-6, TNF-α,) and anti-inflammatory (IL-1Ra, IL-4, IL-10) cytokines (Figs. 5, 6, 7) whose activities may be directly or indirectly related to the development of tumour metastases. Indeed, as demonstrated in our previous experiments, inhibition of the growth of artificial pulmonary tumour colonies in BALB/c and C57BL/6 mice by whole-body, low-level exposures to X-rays coincided with the significantly up-regulated production of IFN-γ, IL-1β, IL-2, IL-12, and TNF-α by NK lymphocytes and activated macrophages obtained from these animals (Cheda et al. 2008). Similar discrepancy between the effects of external low-LET irradiation and internal contamination with HTO was reported by Flegal et al. (2013) who failed to detect any radioadaptive response in mice exposed to low absorbed doses of tritium β-particles (0.0096, 0.96, and 20.8 mGy) via ingested water, while such a response was triggered by low-dose external γ-rays.

Notably, similar to the earlier results obtained by our group in studies of functions of NK-type splenocytes and peritoneal macrophages in BALB/c and C57BL/6 mice externally exposed to X-rays (Cheda et al. 2004b, 2009; Nowosielska et al. 2006b, 2011, 2012), the magnitude of cytotoxic activities of macrophages and NK cells as well as the levels of IFN-γ produced by the latter cells after internal contamination with HTO were not consistent over the time of observation and exhibited wave-like kinetics (Figs. 3, 4, 5, 6). Currently, we have no explanation for these observations and future studies are needed to unveil the mechanism(s) of the cyclic nature of such responses.

In the present investigation, we did not detect any significant differences between the numbers of spleen and bone marrow cells obtained from the HTO-treated and untreated BALB/c and C57BL/6 mice (Table 2). These results are consistent with our finding of no significant impact of the contamination with HTO on the numbers of circulating leukocytes, erythrocytes, and platelets (data not shown). Similar findings were reported by other authors, although the doses used and the timing of the exposures to tritium may differ from the ones employed in the present study. Indeed, as demonstrated by Bannister et al. (2016) who fed pKZ1 transgenic mice HTO, leading to calculated whole-body radiation-absorbed doses of 0.01–180 mGy, no significant changes were detectable in relative spleen weights between the tritium-contaminated and uncontaminated animals at 1 and 8 months after the ingestion of the radioisotope. Also, other researchers demonstrated that application of HTO in drinking water to C57BL/6J mice, with calculated cumulative absorbed radiation doses of 0.0096, 0.96, and 20.8 mGy, did not result in any significant stimulation of apoptosis in splenocytes (Flegal et al. 2013).

In conclusion, the present investigation demonstrates that short-term exposure of both radiosensitive and radioresistant mice to low doses (0.01 or 0.1 Gy) of the tritium-emitted β-particles stimulates cytotoxic activities of NK lymphocytes and activated macrophages, rather than being immunosuppressive. However, in contrast to the results of our previous studies conducted on the same strains of mice exposed to external X-rays, in the present investigation the enhanced NK- and/or macrophage-mediated cytotoxic functions was not associated with inhibition of the development of the injected cancer cell-related neoplastic colonies in the lungs of these animals. Notably, exposures to low and intermediate (1.0 Gy) doses of the HTO-derived radiation did not significantly change the numbers of cells in the bone marrow, spleen, and peripheral blood of the animals from both strains, indicating that such exposures are not likely to adversely affect the haematopoietic system of both radiosensitive and radioresistant mice. The obtained results supplement and expand the existing body of information about biological effects of short-term exposures to low and intermediate doses of HTO-derived low-LET β radiation. However, further studies are needed to more clearly define the relationship between the internal deposition of tritium and a possible immunosuppressive (and cancer facilitating) effect of such a contamination.
